# A novel passive shoulder exoskeleton for assisting overhead work

**DOI:** 10.1017/wtc.2023.1

**Published:** 2023-03-02

**Authors:** Shuo Ding, Anaya Reyes Francisco, Tong Li, Haoyong Yu

**Affiliations:** Department of Biomedical Engineering, National University of Singapore, Singapore, Singapore

**Keywords:** exoskeleton, low weight on arms, overhead work, shoulder injuries

## Abstract

Shoulder exoskeletons (SEs) can assist the shoulder joint of workers during overhead work and are usually passive for good portability. However, current passive SEs face the challenge that their torque generators are often attached to the human arm, which adds a significant amount of weight to the user’s arms, resulting in additional energy consumption of the user. In this paper, we present a novel passive SE whose torque generator is attached to the user’s back and assists the shoulder joint through Bowden cables. Our approach greatly reduces the weight on the user’s arms and can accommodate complex shoulder joint movements with simple and lightweight mechanical structure based on Bowden cables. In addition, to match the nonlinear torque requirements of the shoulder joint, a unique spring-cam mechanism is proposed as the torque generator. To verify the effectiveness of the device, we conducted a usability test based on muscle activations of 10 healthy subjects. When assisting overhead work, the SE significantly reduced the mean and maximum electromyography signals of the shoulder-related muscles by up to 25%. The proposed SE contributes to further research on passive SE design to improve usability, especially in terms of reducing weight on human arms.

## 1. Introduction

Shoulder injury is one of the most common work-related musculoskeletal disorders (MSD) and the average recovery time for shoulder injuries is up to 23 working days (Snodgrass, 2011; Williams and Horodnic, 2019). Consequently, besides the effects on workers themselves, shoulder injuries also place an enormous financial burden on workers, their employers, communities, and the economy (Weale et al., 2018). During overhead work, which is common in the construction, manufacturing, and agriculture sectors, workers are prone to injury, as they must maintain prolonged awkward postures while lifting and holding heavy hand tools or heavy materials above shoulder height (Nappo, 2019). As the arm is raised overhead, the space beneath the acromion (between the acromion and the upper surface of the humerus) narrows and, as the shoulder muscles tire, their ability to hold the head of the humerus and the glenoid fossa of the scapula together decreases. This further reduces the space beneath the acromion, which increases the risk of supraspinatus muscle tendon rupture and leads to severe shoulder pain and injury (Shin et al., 2012). With the goal of reducing workers’ exposure to high strain, devices that can reduce the moment load on the shoulder joint in overhead work have been studied extensively.

A shoulder exoskeleton (SE) is a wearable device that can reduce the burden on shoulder by providing assistive torque to the shoulder (Voilqué et al., 2019). As an on-body device, the SE is more acceptable to workers than off-body devices, which have lower levels of flexibility and often require large-scale modifications of the workplace (Hyun et al., 2020). To assist overhead work, the SE should meet the following requirements. First, the SE must be portable and lightweight to prevent burdening the user’s body (Hyun et al., 2019; Gull et al., 2020). Second, the weight on human arms, which refers to the total weight of the structure moving with the arm, should be particularly emphasized to be low (Sanjuan et al., 2020), since the weight on human limbs (which are far from the center of mass of human) can significantly increase the energy consumption of human in all kinds of motions involving limb movements (Browning et al., 2007; De Santis et al., 2008). In addition, the low weight on arms is especially important for the passive device which does not have an actuator to compensate for its effect, making the assistive torque from the torque generator less effective. Third, the SE must satisfy the nonlinear torque requirements of the shoulder joint and provide maximum assistance when the arm is raised (around 90°) to perform overhead work (Van Engelhoven et al., 2018; Van Engelhoven and Kazerooni, 2019).

Depending on the choice of the torque generation, SEs can be defined as active and passive (or semi-passive) (McFarland and Fischer, 2019; Crea et al., 2021). An active SE employs motors or pneumatic actuators to generate adequate assistive torque based on sensor information (Ebrahimi, 2017; Blanco et al., 2020; Zhou et al., 2021). However, due to the weight of the actuators, power sources, and complex transmission mechanisms, active SEs are often heavy with poor portability, which has limited their application. Passive SEs, on the other hand, generate assistive torque by storing potential energy in elastic elements (commonly springs) and using changes in arm elevation to transform it into assistance. Currently available passive SEs include ShoulderX by SuitX (Van Engelhoven et al., 2019), MATE by IUVO (Pacifico et al., 2020), AirFrame by Levitate Technologies, Inc. (Spada et al., 2017), and EksoVest by EksoBionics (Alabdulkarim et al., 2019; Kim and Nussbaum, 2019). Laboratory prototypes include the Hyundai Vest Exoskeleton (H-VEX) (Hyun et al., 2019) and a semi-passive upper-limb exoskeleton based on MATE (H-PULSE) (Grazi et al., 2020). However, as shown in Table 1, most of these devices attach their torque generators to the human arm, which causes the drawback of adding large weight to the user’s arms. In an attempt to mitigate this problem, the passive SEs Skelex 360-XFR by Skelex (de Vries et al., 2019) and PAEXO by Ottobock (Maurice et al., 2019a integrate their torque generators into the support links that are parallel to the torso, but the support links (including the torque generators) are still being moved by the user’s arms, which consumes energy. The SE developed by Rossini et al. (2021) uses a remote actuation system to transmit force via cables. However, the cable transmission mechanism includes multiple heavy and bulky rotational joints, which greatly increases the weight moving with the user’s arm.

**Table 1.** Current passive shoulder exoskeletons

Name	Total weight (kg)	Assistive torque (Nm)	Torque generator position
ShoulderX (Van Engelhoven et al., 2019)	3.17	10	Attached to human arms
MATE (Pacifico et al., 2020)	3.5	5.5	
Airframe (Spada et al., 2017)	2.7	NA	
EksoVest (Kim and Nussbaum, 2019)	4.3	13	
H-VEX (Hyun et al., 2019)	2.5	10	
H-PULSE (Grazi et al., 2020)	5	5.5	
Skelex (de Vries et al., 2019)	2.5	6.1	On support links, moved by human arms
Paexo (Maurice et al., 2019a	1.9	NA	
SE by Rossini et al. (2021)	3.8	3	Cable transmission mechanism moved by human arms
Proposed SE	3.2	10	Fixed on human back

To overcome the above challenges, in this paper we present a new passive SE for assisting overhead work. A novel actuation approach is proposed in which the torque generator is fixed on the user’s back and drives the shoulder joint through Bowden cables. The actuation reduces the weight on human arms and the use of Bowden cables provides a flexible connection allowing the shoulder hinge to move with human shoulder while being actuated. In addition, we have designed a unique spring-cam mechanism that can meet the nonlinear torque requirements of the shoulder joint. The SE prototype weighs 3.2 kg, of which only 0.2 kg is moved by the human arm on each side, while providing a maximum torque assist of 10 Nm to the user’s shoulder during overhead work. To verify the feasibility and efficacy of the device, we evaluated the effect of the use of the exoskeleton on shoulder muscle activity in healthy subjects during simulated industrial overhead tasks.

The paper is structured as follows. Section 2 presents the details of the design of the implemented SE. Section 3 describes the experiments performed to validate the proposed device. The results of these experiments are discussed in Section 4. Finally, Section 5 concludes the paper.

## 2. Method

### 2.1. Working principle of the SE

We based the SE torque profile on a simplified bio-mechanical model of the shoulder moment considering the corresponding weight of the upper arm, forearm, and hand, as well as the tool being handled (shoulder flexion in sagittal plane, as proposed by Van Engelhoven and Kazerooni (2019)). During overhead work, a worker raises the upper arm to a certain angle while holding a tool in the hand, as shown in Figure 1(a). Then the torque of the upper limb joints ( 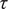 ) can be given by(1)

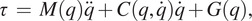

where 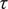 is a 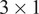 vector of the torque (the torque of shoulder, elbow, and wrist joints, 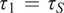 representing the shoulder joint torque), 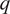 is a 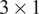 vector of joint positions (the angles of shoulder, elbow, and wrist joints, 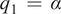 representing the shoulder joint angle), 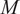 is the 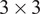 inertial matrix, 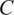 is the 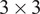 matrix that represents Coriolis/centrifugal terms, and 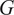 is the 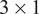 gravity vector.

**Figure 1.**
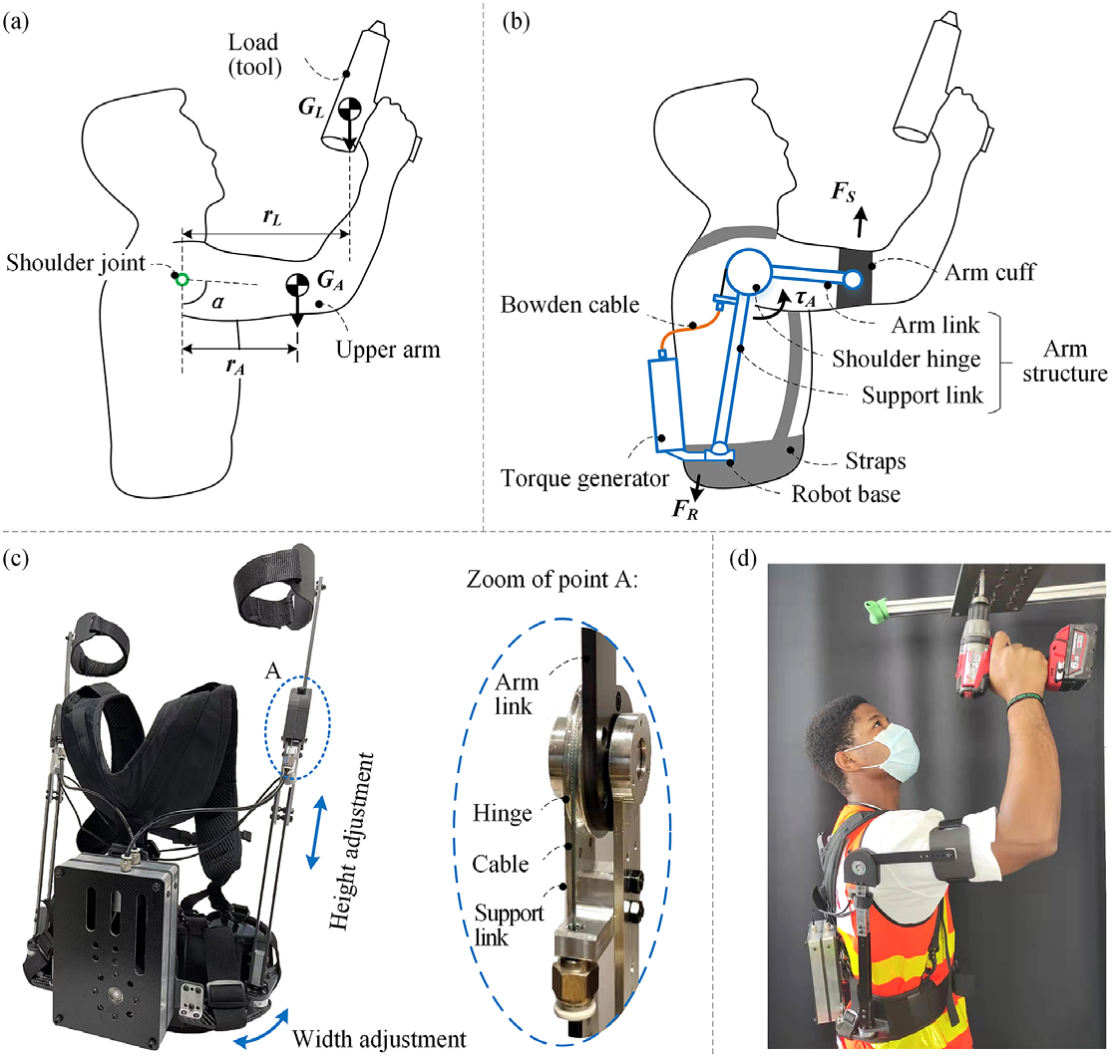
Overview of the proposed SE. (a) Force analysis of overhead work (shoulder flexion in the sagittal plane). (b) Working principle of the proposed SE. (c) SE prototype and structural details. (d) A user wears the SE to work.

We can further simplify (1) by assuming that the arm is in a static or quasi-static condition, and using 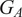 to represent the equivalent gravity of the arm, then the shoulder joint torque should meet the following requirement:(2)

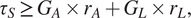

where 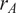 is the distance between the shoulder joint and the equivalent center of mass of the arm, 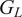 is the weight of the tool, and 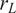 is the distance ( 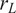 ) between the shoulder joint and the tool being handled.

Since the moment arms of 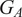 and 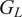 ( 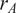 and 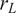 ) are much larger than those of the shoulder musculature, the shoulder muscles must maintain high tension to generate sufficient torque and are prone to fatigue. SEs are used to reduce the muscle activity in the shoulder region by creating a support force that acts in the opposite direction to 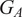 and 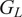 . As shown in Figure 1(b), the device generates an assistive torque ( 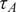 ) on the shoulder hinge and supports the upper arm ( 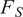 ). Then, the moment load on the shoulder joint is reduced by(3)

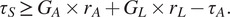



As the SE releases the shoulder joint, it transfers the load to the robot base resulting in the reaction force, 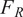 . Since the robot base is attached to the user’s body by straps, the load is distributed to the waist and upper body (e.g., chest and upper back). Soft straps with a large contact surface are used to reduce the strain on the user body.

### 2.2. Overall structure of the SE

According to the analysis of previous studies about overhead work, we conclude the detailed design requirements of the SE as follow. First, the weight of the SE should be targeted at 3 kg (Hyun et al., 2019). Second, the weight on human arms must be as small as possible (Rossini et al., 2021). Third, the SE should provide the full range of motion (ROM) of the shoulder joint (Voilqué et al., 2019). In addition, the SE should generate an effective assistive torque profile that can match the nonlinear torque load of the shoulder joint. In this work, by using Bowden cables, we made the device lightweight with low weight on human arms and can permit nearly full ROM of the shoulder joint (Table 2). A spring-cam mechanism was designed to meet the assistive torque requirement. As shown in Figure 1(b), the proposed SE prototype mainly consists of four parts, that is, a base, a torque generator, and two arm structures (left and right side). The base is attached to the human back by straps and the torque generator is fixed to it. Each of the arm structures includes an arm link, a support link, and a shoulder hinge. One end of the arm link is attached to the human upper arm by an arm cuff and straps while the other end is fixed to the shoulder hinge. The shoulder hinge is located close to the center of human shoulder joint and is rotationally coupled to the support link. Two elastic ropes are added between the support link and the back straps to stabilize the support link when not being worn by the user. The Bowden cable transfers the force from the torque generator to drive the shoulder hinge (with a diameter of 50 mm). The structural details of the shoulder hinge are shown in Figure 1(c), zoom of point A.

**Table 2.** Measured ROM of the shoulder joint with and without the SE (mean of five trails)

Shoulder movement	ROM without the SE	ROM with the SE
Flexion	155.16° ± 4.88°	150.72° ± 2.97°
Extension	45.22° ± 2.57°	34.88° ± 3.63°
Abduction	140.48° ± 2.53°	136.59° ± 0.43°
Adduction	36.02° ± 5.45°	17.23° ± 1.84°
External rotation	77.18° ± 3.45°	74.99° ± 3.37°
Internal rotation	64.62° ± 1.47°	55.79° ± 1.83°

The structural parts of the SE are made of carbon fiber and aluminum, which have sufficient strength and smaller density compared to steel. The non-structural parts (such as the shells on shoulder hinges) are 3D-printed with ABS plastic. In addition, we verified the strength of the structure by finite element analysis. The height and width adjustment structures make the SE adaptable to different body shapes (1.65–1.85 m). A user wearing the SE for overhead work is shown in Figure 1(d).

**Figure 2.**
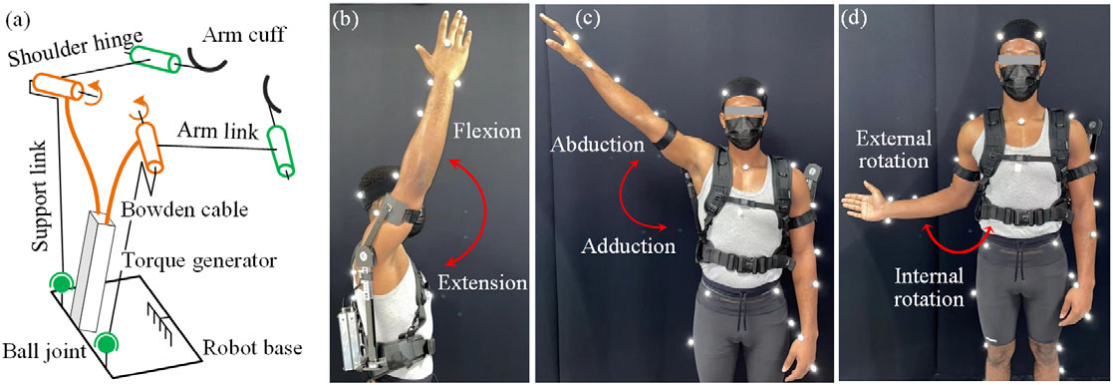
(a) Degree of freedom of the SE. The joints in green color are passive. The joints in orange color are active. (b) Measurement of the ROM of the shoulder joint with the SE. Flexion and Extension. (c) Abduction and adduction. (d) External rotation and internal rotation.

The mechanical design of the SE is challenging due to the multiple degree of freedoms (DOFs) of human shoulder (Braman et al., 2009). The exoskeleton should be flexible enough to prevent obstructing the user during complex shoulder movements, which causes discomfort or even injury. In this work, instead of adding many constraints to fix the shoulder hinge to a certain position relative to human shoulder, we give the shoulder hinge sufficient flexibility to follow the complex movements of human shoulder by taking the advantage of the Bowden cable. The DOF of the exoskeleton is shown in Figure 2(a). The support link is connected to the robot base through a ball joint, which allows the whole arm structure to follow the movements of the human arm. The rotation DOF between the arm link and the arm cuff is also helpful to reduce the constraints on the arm. More importantly, the use of Bowden cables provides a flexible connection between the torque generator and the shoulder hinge, allowing the shoulder hinge to move with human shoulder while being actuated. As reported by Voilqué et al. (2019), our arm structure (similar to PAEXO Maurice et al., 2019a) is one of few that can give mobility for the center of rotation of the shoulder joint to minimize the constraint on human arm. To test the flexibility of the exoskeleton, we measured the ROM of the shoulder joint of one healthy subject with and without the SE by using a 3D motion capture system as shown in Figure 2(b)–(d). Kinematic data were recorded with a 8-camera, three-dimensional motion capture system (Vicon Vero V2.2 IR; Vicon Motion Systems, Oxford, United Kingdom). Thirty-eight retro-reflective markers (14-mm diameter) were placed on landmarks following the Plug-in-Gait full-body market placement model. Captured data were sampled at 100 Hz. In the experiment, the subject was told to move the arm to the limit of his ROM in the directions of flexion/extension, abduction/adduction, and external/internal rotation. As reported in Table 2, The ROM with the SE in three directions is 92.6%, 87.2%, and 92.2% of that without the SE, indicating the SE is able to reach almost the full range of the shoulder motion before colliding with the user.

### 2.3. Torque generator design

**Figure 3.**
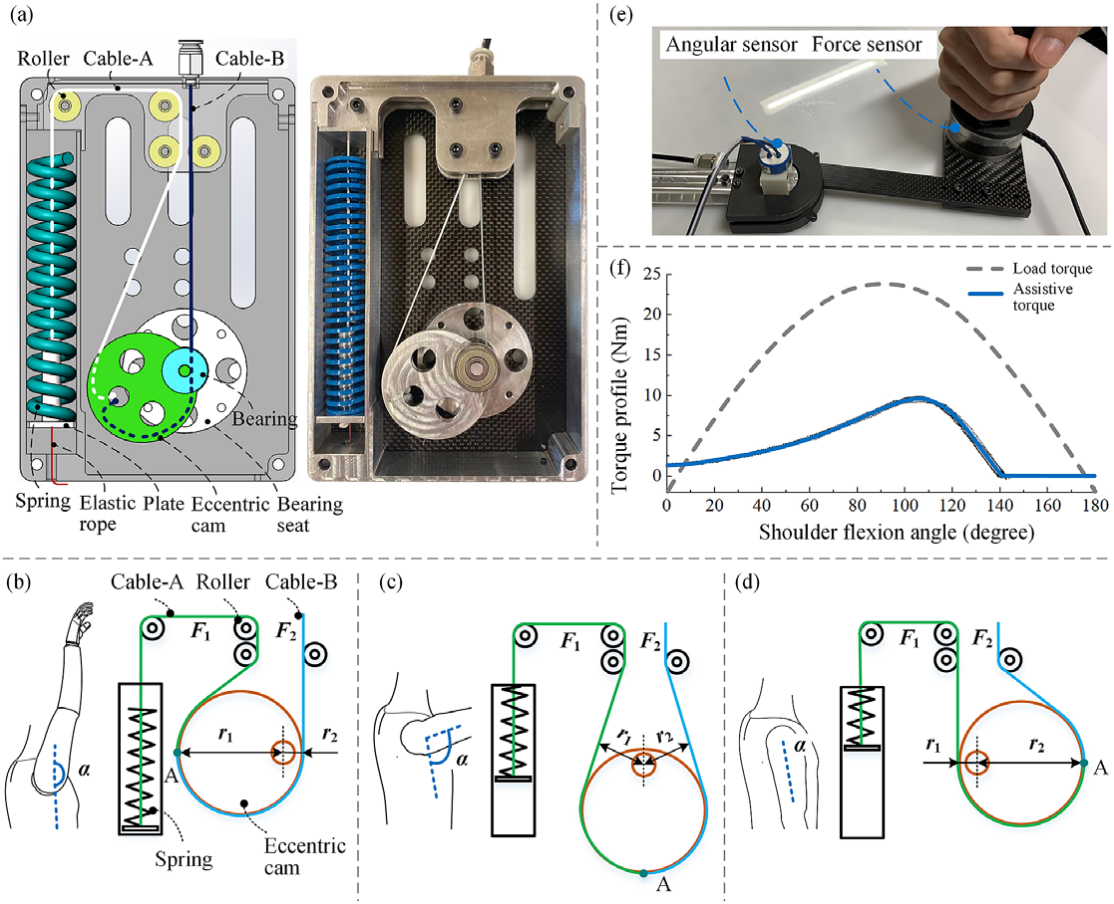
Design and working principle of the spring-cam mechanism. (a) The structure of the spring-cam mechanism. (b) When the arm is raised up ( 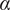 is close to 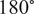 ), the spring is not compressed ( 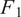 is zero) and the force of cable B ( 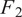 ) is zero. (c) If 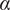 is around 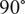 , the spring is compressed ( 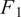 is large), since the force arms ( 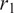 and 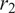 ) are almost equal, 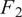 is also large. (d) When the user puts down the arm ( 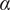 is around zero), the spring is fully compressed ( 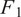 reaches the maximum value), however since 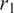 is much smaller than 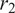 , 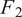 can be small. (e) Test bench to validate the assistive torque output. (f) Assistive torque of the torque generator (and the load torque on shoulder) as a function of the shoulder flexion angle. The load torque is estimated by Van Engelhoven et al. (2019) for 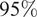 males (arm mass 6.2 kg) holding a tool of 2.25 kg (close to the tool weight in our experiments).

When the arm is raised from the natural downward position ( 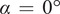 ) to the highest overhead position ( 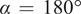 ), the moment arm of gravity ( 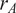 and 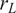 , Figure 1) first increases and then decreases, with the largest moment occurring when 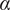 is around 90°. Therefore, the moment load of the shoulder is not linear with respect to the joint angle. Based on the torque requirement of the shoulder joint, we design a spring-cam mechanism to generate adequate force on Bowden cables. The structure of the spring-cam mechanism is shown in Figure 3(a). The mechanism is mainly composed of an eccentric cam, a compression spring, cables, and rollers. The eccentric cam is rotatably coupled with the bearings supported by the bearing seats. One end of a cable (cable A) is stuck to the eccentric cam and the other end is attached to a plate that is fixed with one spring. The plate has a cylindrical structure that can guide the spring. The square cavity where the spring is placed can also guide the spring motion. Another cable (cable B) is connected between the eccentric cam and the shoulder hinge, passing through the cable sheath. The directions of the cables are guided by rollers that are supported by the shell of the torque generator. In order to ensure the cables are always tight, an elastic rope is attached between the plate and a fixed point on the shell.

The working principle of the spring cam is based on the force balance relation of two cables wound on an eccentric cam (both fixed at point A), as shown in Figure 3(b)–(d). Cable A is connected to a compression spring to generate the force and cable B is connected to the shoulder hinge to drive it. Let 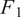 and 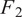 represent the force on cable A and cable B, respectively. Then, the relation between 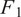 and 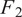 can be written as follow (here we use a simplified model that only considers the force relation in a static or quasi-static condition, assuming no friction):(4)

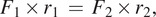

where 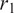 and 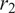 represent the moment arm of 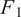 and 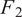 , respectively.

As shown in Figure 3(b), when the arm is at the highest position ( 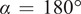 ), the spring is not compressed. 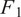 and 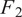 are close to zero (with small preload) and no assistive torque is generated. When the user lowers the arm, cable B is pulled and rotates the eccentric cam. In this way, the spring is lifted and compressed.

The device outputs large torque when 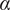 is around 90°. As shown in Figure 3(c), the spring is compressed and 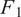 is large. Since 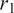 is almost equal to 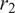 , 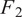 is close to 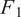 and is also large.

If the user puts down the arm, the spring is fully compressed and 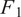 reaches the maximum value (Figure 3(d)). However, at that point, 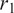 is much smaller than 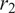 , so 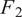 is much smaller than 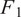 . The device generates small assistive torque to the user. The mathematical model of the mechanism is shown in Appendix.

To validate the torque output of the device, we set up a test bench as shown in Figure 3(e). The support link was fixed on a table and the arm link was controlled by human hand. A six-dimensional force sensor (ATI, Mini58, USA) was installed on the arm link to measure the interaction force, and an angle sensor was coaxially coupled with the shoulder hinge to measure the angle. In the test, the operator held the grip and moved the arm link very slowly (quasi-statically). The interaction force and shoulder flexion angle were recorded synchronously. Then, the assistive torque was calculated using the interaction force. When the spring was not compressed (from 145° to 180°), the torque was recorded as zero by default. The signals of the force sensor and the angle sensor are acquired by a dSPACE 2002 ADC board with a sampling rate of 1000 Hz and then filtered by a 25 Hz low-pass filter.

The assistive torque (and the load torque on shoulder) as a function of the shoulder flexion angle is shown in Figure 3(f). The load torque is estimated by Van Engelhoven et al. (2019) for 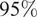 males (arm mass 6.2 kg) holding a tool of 2.25 kg (close to the tool weight in our experiment). The device provides small assistive torque (about 1 Nm) near 0° and large assistive torque between 90° and 120°, which is consistent with the trend of the load torque. Currently, the maximum assistive torque is approximately 10 Nm by using a spring with the stiffness of 13.5 N/mm. We selected the maximum assistive torque of 10 Nm (which is smaller than the maximum load torque 24 Nm) for the following reasons. First, the SE aims to help release the shoulder muscle but not remove all the load torque on the shoulder. Second, for a passive SE, the assistive torque should not be too large to make it difficult for the user to lower the arm. Third, the results by Van Engelhoven et al. (2019) indicated that 10 Nm is a preferred assist level regardless of the task type and tool weight. When using different springs, we can adjust the maximum torque output of the SE since different users may still prefer different percentages of the arm gravitational torque.

## 3. Experiments

To validate the effectiveness of the proposed SE, we conducted a series of evaluation tests that simulated the industrial overhead work with a hand drill. The experiments took place at the BioRobotics Lab at the National University of Singapore. Ten healthy male subjects (age: 27 ± 4; height: 172 ± 5 cm; weight: 70 ± 10 kg; all right-handed) voluntarily participate in the experiments. No participant reported any pre-existing musculoskeletal shoulder injury at the time of data collection. The experimental protocol was approved by the Institutional Review Board of the National University of Singapore (NUS-IRB study H-20-027) and before the start of the trial, the authors explained the general purpose of the study and obtained oral and written informed consent from the participants.

### 3.1. Experiments set up

**Figure 4.**
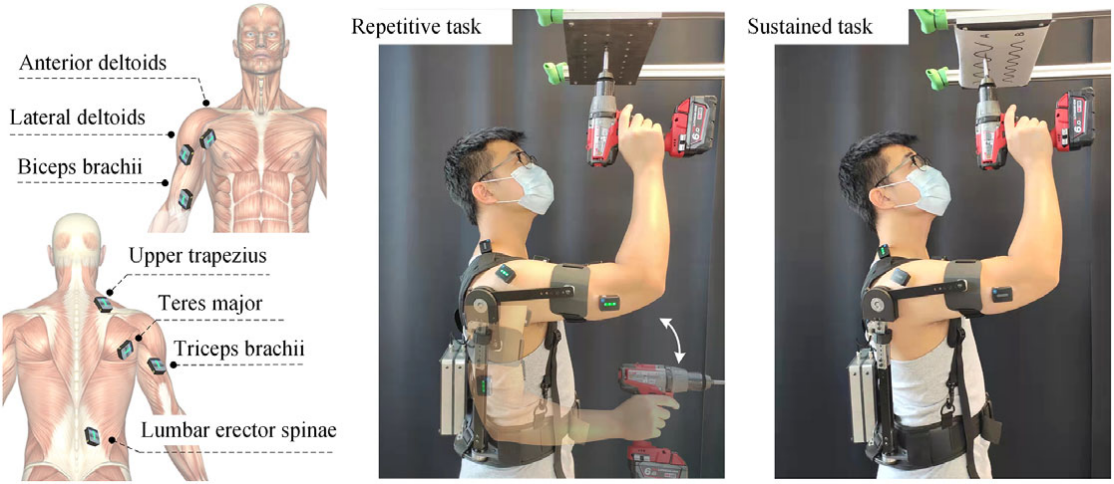
Positions of the EMG sensors and the simulation test of industrial overhead work (repetitive task and sustained task).

Repetitive overhead and sustained tasks were simulated to be representative of possible applications of the SE, from which muscle activations were recorded using surface electromyography (EMG). The simulation tests were performed following the guidelines outlined in the previously published study by Van Engelhoven et al. (2019). Task types were performed in random order, and then the order of exoskeleton and non-exoskeleton conditions was alternated between participants to counteract the effects of learning and/or fatigue. For each task type, subjects used a battery-powered drilling tool weighing 2.3 kg. The study tasks were performed with participants standing in front of an aluminum rung connected to a height-adjustable work piece that had twenty-five equally spaced holes of 1.6 cm in diameter. The height of the work piece was adjusted to the individual overhead working height (with shoulder and elbow flexed at 90° when the dominant hand is placed in the workspace). A repetitive task involved inserting a series of screws with the battery-powered drill, which required the subject to alternate between raising the dominant arm toward the work piece and lowering it to grab the next screw from a waist-mounted tool bag. A sustained task required drawing a series of sinusoidal lines held against the work piece, which required a constant 90° angle of flexion of the shoulder of the dominant arm, as the elbow and wrist were used to follow the lines with the tip of the drill. Participants were instructed to perform the overhead work tasks without strongly gripping the drilling tool with the right hand, as shown in Figure 4, and completed two 45 s trials for each task type (i.e., either sustained or repetitive). Participants had at least 5 min of rest between each trial and rested at least 15 min between each of the two types of tasks. If a participant indicated muscle fatigue, a rest of sufficient duration was taken to fully recover. The entire testing session lasted about 3 hr.

Muscle activity was recorded using the Trigno Wireless Avanti system (Delsys, Inc., USA). EMG signals were measured from seven muscles: anterior deltoid (AD), lateral deltoid (LD), upper trapezius (UT), biceps brachii (BB), triceps brachii (TB), lumbar erector spinae (LES) at the level of the L3 vertebrae and teres major (TM), as shown in Figure 4. Electrodes were kept in place between tests with and without the exoskeleton. Before applying the electrodes, the skin was cleaned with alcohol. Participants then performed three 3-s maximal voluntary contractions (MVCs) for each of the muscles studied (according to the SENIAM protocol Hermens et al., 2000) and were verbally encouraged to produce as strong a contraction as possible. Shoulder, neck, and upper back muscles were used to evaluate the effectiveness of the SE in relieving shoulder muscles during overhead tasks based on a comparison of two cases with/without use of the exoskeleton. The lower back and trunk muscles were evaluated to determine the impact of the SE on the spine, as previous devices have revealed increased muscle activations, which in turn might contribute to the development of other MSDs, such as back injuries. EMG data were sampled at 2,000 Hz. After recording, the EMG signals were full wave rectified and smoothed using an RMS filter (125 ms window size). The averaged RMS signals were then normalized with respect to the initial MVC value for each muscle, and time-averaged and maximal activation values were calculated from the normalized data. Due to the small number of test subjects and the repeated-measures design of the study, simple paired *t*-test was used to compare the condition with the SE versus the condition without the SE, with 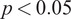 considered statistically significant. Statistical analyses were performed using GraphPad Prism software version 9.0 (GraphPad Software, San Diego, CA, USA).

### 3.2. Experimental results

**Figure 5.**
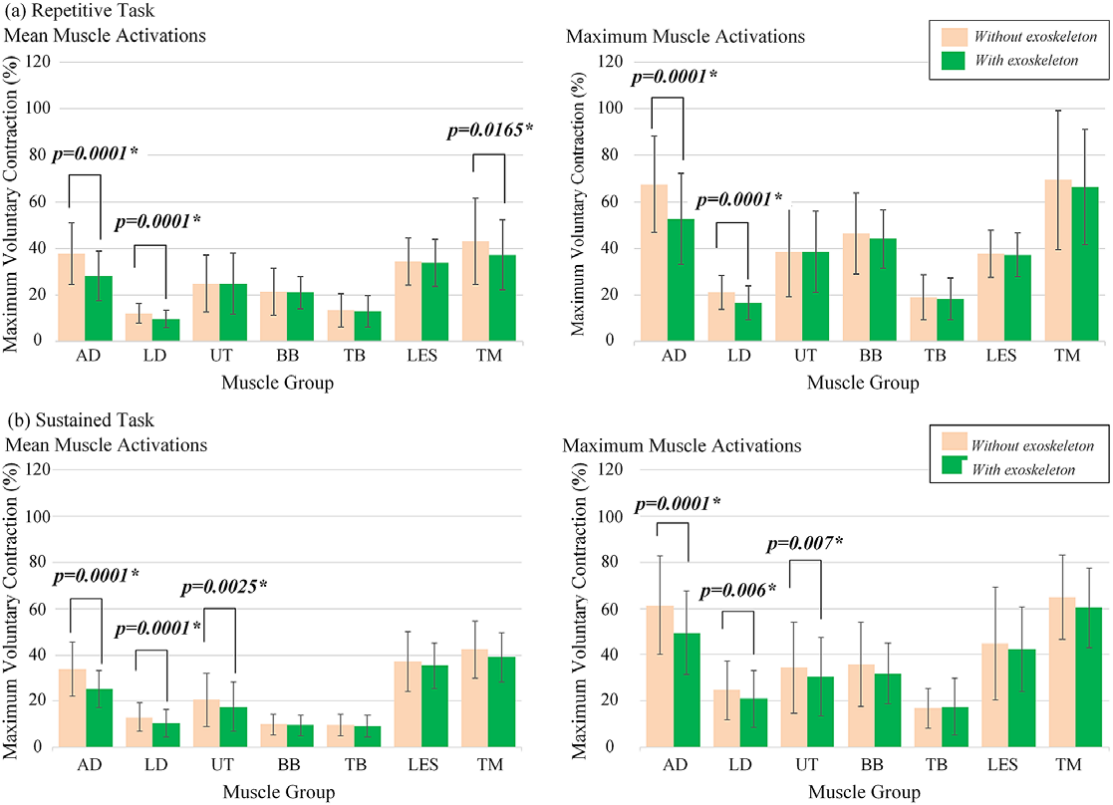
Statistical results of experimental tasks. (a) Repetitive overhead simulated task. (b) Sustained overhead simulated task. The measured muscles of right upper extremity were anterior deltoids (AD), lateral deltoids (LD), upper trapezius (UT), biceps brachii (BB), triceps brachii (TB), lumbar erector spinae (LES), and teres major (TM). The error bars indicate standard deviation intervals, and the symbol 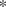 indicates a significant difference between the experimental groups.

The results of the simulation tests are presented in Figure 5. In both repetitive and sustained tasks, it was confirmed that the use of the passive SE resulted in a significant reduction of EMG activation (as a percentage of the MVC) of the muscles responsible for shoulder flexion (i.e., anterior deltoid and lateral deltoid). Furthermore, although some significant changes in TM and UT muscle activations were also found for some of the simulated tasks, there were no significant differences in mean or maximal activations of BB, TB, or LES for any of the experimental conditions.

Overall, during the simulated repetitive and sustained tasks, mean AD and LD activations were consistently lower for the condition with SE than for the condition without SE ( 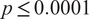 ). A similar trend was observed for maximal AD and LD activations ( 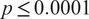 ). Interestingly, lower mean TM activations were observed with the use of the SE during the repetitive task ( 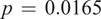 ), but this effect was not observed in the sustained task. In contrast, it is observed that when participants performed the sustained overhead sustained task while wearing the SE, the mean ( 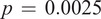 ) and maximum ( 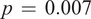 ) muscle activation of the UT decreased similarly to that of other muscles (i.e., AD and LD), but this effect was not observed in the repetitive task.

In summary, the effectiveness of the SE in releasing the shoulder musculature during overhead tasks is represented in Table 3, which is the list of the percentage reduction of muscle activation when using the SE observed in the overall experiment. The change in muscle activation related to the condition without SE showed a reduction of up to 25% for AD activations and up to 21% for LD during the repetitive and sustained tasks. Similarly, UT reduced its mean and maximal activations by up to 14% while maintaining a sustained lifting posture overhead.

**Table 3.** Reduction percentage (%) of muscle activity by each muscle when wearing the SE during the overhead simulated task

	AD	LD	UT	BB	TB	LES	TM
Repetitive task							
Mean	25.52	20.09	0.00	1.40	4.12	1.72	13.40
Max	22.03	21.59	0.32	4.88	3.65	1.48	4.43
Sustained task							
Mean	25.50	18.39	14.67	4.09	4.10	4.82	7.56
Max	19.32	14.80	11.73	10.76	−4.28	5.28	7.27

## 4. Discussion

### 4.1. Design Innovations

When developing the SE for overhead work assistance, we considered both controlling the self-weight of the SE and the added weight to the human arm (the weight of the arm structure). The small self-weight is necessary for assistive wearable devices to move with the user. Our prototype weighs 3.2 kg, which is close to the target weight of 3 kg (Hyun et al., 2019). As shown in Table 1, although not the lightest, the proposed SE is as heavy as the commercial passive SE ShoulderX (Van Engelhoven et al., 2019) and is lighter than MATE (Pacifico et al., 2020) and EksoVest (Kim and Nussbaum, 2019). In addition to the use of Bowden cables, the unique torque generator design is also helpful to control the weight and size of the device. Compared to the cam mechanism presented by Asgari et al. (2020), our mechanism is more compact and lighter by applying a single eccentric-wheel structure rather than the two-wheel structure (one variable radius cam wheel and one fixed radius wheel Asgari et al., 2022). In addition, our mechanism has larger range of movement (the range of the mechanism by Asgari et al., 2020) is only 0°–90° in sagittal plan), which is more suitable for overhead work.

Reducing the weight of the “end effector” attached to human limbs is important for exoskeletons to save energy for the user (also motor energy for active exoskeletons). For this reason, many lower limb exoskeletons use Bowden cables to transmit force and eliminate the need to place the torque generator along the legs (Aguirre-Ollinger et al., 2019; Hidayah et al., 2020; Welker et al., 2020). Compared to the lower limbs, the upper limbs are more flexible in their movements. In addition, the upper limbs are more sensitive to the mass attached to them, as they are much lighter than the lower limbs. Therefore, the low weight on human arms is of particular importance to ensure user comfort and save their strength during the long-term use. In this work, we achieved a weight of the arm structure (0.2 kg, single side), which is only 6% of the total weight of the device. In particular, the arm link (and cuff) between the shoulder hinge and the upper arm weighs 0.08 kg and generates around 0.1 Nm gravity moment on the shoulder joint, which is far less than the assistive torque. The lightweight arm link achieves good transparency between 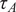 and 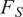 (see in Figure 1(b)), which is important for the user to obtain an adequate support force on the upper arm, since there are no sensors attached to the human body to measure the human-robot interaction force.

At the shoulder hinge, the SE produces a maximum assistive torque of 10 Nm when the shoulder flexion angle is 105° (as shown in Figure 2(f)). This assistive torque profile is similar to that of the SE H-VEX (Hyun et al., 2019). In fact, by setting different initial angles of the shoulder hinge ( 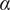 ), we can shift the assistive torque curve horizontally and adjust the angle corresponding to the maximum torque for specific requirements. In addition to adapting to the moment load on the shoulder joint, the torque profile that provides a small assistive torque when the arm is lowered or raised too high is also useful for ensuring user comfort and safety (Van Engelhoven et al., 2018). When the user lowers the arm to rest or perform other daily activities (e.g., a worker finishes overhead work in one location and walks to another, or sits down to rest), the device outputs a small torque that can allow the arm to move freely for comfort. If the user raises the arm too high (e.g., shoulder flexion angle exceeds 140°), the small assistive torque can prevent the device from pushing the arm into a hyperflexed position for safety.

### 4.2. SE effectiveness

The simulated overload tests aimed to examine the “expected” effects of using the SE in terms of muscle activations of the superficial muscles responsible for shoulder flexion. Overall, our findings indicate that the use of the current prototype successfully reduced the mean and maximal activations of the deltoid muscle groups by up to around 25% and 21%, respectively, which are the main muscles involved in performing shoulder flexion. Similar results were found with the passive SEs ShoulderX (Van Engelhoven et al., 2019), PAEXO (Maurice et al., 2019a), and MATE (Pacifico et al., 2020), where the activity of the deltoid muscles during manual lifting and lowering tasks was significantly reduced. The lack of significant differences between task types in unloading the deltoid muscle groups indicates that our SE may be equally applicable to static (sustained) or dynamic (repetitive) tasks. Therefore, such a reduction may help to reduce the possibility of injury.

In addition to the positive, yet expected, results in the deltoid muscles, we observed significant EMG reductions in the UT muscles during overhead sustained tasks of up to 14%. This effect was also demonstrated in a previous study (Van Engelhoven et al., 2018), in which the authors reported a 15% reduction in UT activations when the ShoulderX was used when handling a light tool overhead. The reduction in UT activations during sustained tasks could be explained by the fact that this muscle primarily contracts to maintain scapula in a relatively static position during arm elevation (Grazi et al., 2020). Reductions in these muscles could indicate that the SE reduced the muscular effort required to stabilize the shoulder when the arms are held elevated for prolonged periods. In addition to maintaining the shoulder in flexion position, it has been suggested that the UT also represents a component of load transfer between the shoulder complex and the spinal (Van Engelhoven et al., 2018). Therefore, the reduction in UT activations during sustained work may indicate a reduction in spinal loading, as the SE is able to transfer reaction forces beyond the shoulders through the base of the SE to the waist and upper body of the user. This effect in reducing spinal loading was confirmed by unchanged muscle activations of the LES muscles when using the device. These results are also similar to those reported by the PAEXO exoskeleton (Maurice et al., 2019a), which may indicate that our SE, like the PAEXO, is able to unload the shoulder without causing additional lumbar loading. This is an important finding, as previous similar devices, such as the H-VEX (Hyun et al., 2019), have reported increased activation of the lower back muscles, which in turn could lead to the development of other MSDs in the long term.

One of the concerns in the design of the SE was the effort the user had to make to lower the arm in the opposite direction of the SE assistive torque (Grazi et al., 2022). Compared to active exoskeletons, passive exoskeletons with spring only provide a supporting moment when the spring is put in tension. As a result, movements in the direction opposite to the spring force may require more effort. However, we did not observe such effects on the antagonist muscles during shoulder flexion (i.e., TB and TM). On the contrary, an interesting finding during repetitive tasks is the reduction in mean TM activations. The reduction of the EMG activity of the TM muscles during repetitive tasks could be explained by the fact that this muscle mainly helps to bring the previously raised arm downward and backward toward extension (Donohue et al., 2017). Therefore, in repetitive tasks in which several extension movements are performed, the reduction in these muscles could indicate that the SE reduced the muscle effort needed to lower the previously raised arm, probably because the moment generated by the SE was smaller than the gravitational moment.

Although the results suggest that users were able to instantaneously modulate their deltoid and upper trapezius muscle activations to the SE assistance without putting additional strain on the user’s back, longer training sessions could potentially enhance the effectiveness of the device.

### 4.3. Limitations and future works

A limitation of the proposed SE is the lack of a maximum torque adjustment mechanism. Although the maximum torque of 10 Nm has been shown to be adequate for most types of overhead work and tool weights (Van Engelhoven et al., 2019), it will be more convenient for the user to adjust the maximum torque according to their needs under specific working conditions. Therefore, in the future, we will add the function of adjusting the maximum torque to the device. Another limitation of the device is the friction caused by the Bowden cable. The friction affects the force transmission efficiency, therefore, we need to select suitable springs and validate the mechanism by measuring the actual assistive torque (Hofmann et al., 2018). The friction of Bowden cable is related to the materials, the bend, the force added to the cable, and so on. Since the material and the target force of the Bowden cable are determined, in the future we will further optimize the bending of the Bowden cable to minimize the curve angle. For experimental validation, it has been demonstrated that SE can effectively release the shoulder muscles. However, the current evaluation was of relatively short duration and for a simple overhead task with subjects inexperienced in performing overhead work. Although novice participation has not been considered a major limitation in most previously published studies, it is possible that the effects of the SE vary from those of skilled workers. Further experiments are still needed to study the impact of systematic adoption of SE on the incidence of shoulder MSDs. Therefore, our future work will investigate the long-term effects of the device on real workers.

## 5. Conclusion

Most of the latest generation of exoskeletons that assist in overhead work integrate bulky and heavy designs in which their torque generators are typically attached to the human arm, adding a significant amount of weight to the user’s arms. In this work, we propose a transmission component that allows the SE torque generator to be installed on the user’s back and drive the shoulder joint remotely through Bowden cables, while maintaining a lightweight arm structure and reducing the added weight to the human arms. During overhead work, the SE provides a maximum assistive torque of 10 Nm with a spring-cam mechanism. We tested the proposed SE on 10 healthy subjects, revealing a significant reduction in muscle activity during repetitive and sustained shoulder flexion tasks.

## Figures and Tables

**Figure 1.**
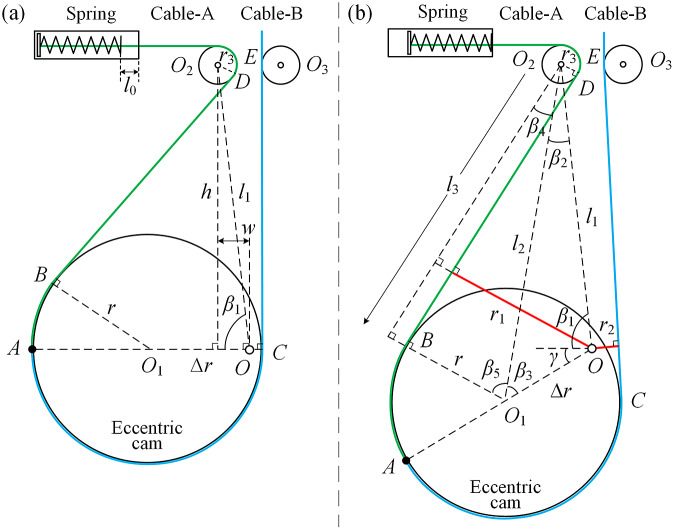
Mathematical model of the spring cam. (a) Initial position of the spring-cam mechanism. (b) Geometric relationship of the mechanism when the eccentric cam rotates counterclockwise by an angle 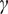 _._

## Data Availability

The experimental EMG data can be made available to interested researchers upon request by email to the corresponding author.

## Appendix

The mathematical model of the spring-cam mechanism is shown in Figure A1 (here we use a simplified model that only considers the force relation in a static or quasi-static condition, assuming no friction, and the unit of the angles in the model is in radians). We use  and  to represent the geometric center and the center of rotation of the eccentric cam, respectively.  and  are the centers of rotation of the two rollers, which are symmetric with respect to the vertical line passing through  .  is the fixed point of the cables on the eccentric cam and  ,  ,  , and  are the tangent points of the cables. We defined the initial position of the mechanism as when  is horizontal and  is to the left of  , as shown in Figure A1(a). The design parameters include the eccentric cam radius  , the eccentricity  , the roller radius  , the vertical and horizontal distance between the roller and the eccentric cam  and  , the extra cavity length  , and the spring stiffness  . The initial parameters  and  are given by

(A-1)

When the eccentric wheel rotates counterclockwise by an angle  , the geometric relationship of the mechanism is shown in Figure A1(b). If  , the moment arm of the force on the cable A (  ) and the displacement of the cable A (defined as the length of  adding the arc length of  , represented by  ) are calculated by

(A-2)

If  ,  and  are calculated by

(A-3)

The force on cable A (the spring force) is obtained according to Hooke’s Law:

(A-4)

where  is the initial value of  (when  ).

Since the two rollers are symmetric with respect to the vertical line passing through  , the moment arm of the force on the cable B (  ) and the displacement of the cable B (defined as the length of  adding the arc length of  , represented by  ) can be calculated according to the symmetry of the structure as

(A-5)

By substituting  ,  , and  into (4), we can get the force on cable B (  ). Then the torque and angle of the shoulder hinge can be calculated by

(A-6)

where  is the radius of the shoulder hinge,  is the initial angle of the shoulder hinge when  , and  is the initial length of cable B.
